# Complete Genome Sequencing of *Mycobacterium bovis* SP38 and Comparative Genomics of *Mycobacterium bovis* and *M. tuberculosis* Strains

**DOI:** 10.3389/fmicb.2017.02389

**Published:** 2017-12-05

**Authors:** Cristina Kraemer Zimpel, Paulo E. Brandão, Antônio F. de Souza Filho, Robson F. de Souza, Cássia Y. Ikuta, José Soares Ferreira Neto, Naila C. Soler Camargo, Marcos Bryan Heinemann, Ana M. S. Guimarães

**Affiliations:** ^1^Laboratory of Applied Research in Mycobacteria, Department of Microbiology, Institute of Biomedical Sciences, University of São Paulo, São Paulo, Brazil; ^2^Department of Preventive Veterinary Medicine and Animal Health, School of Veterinary Medicine and Animal Sciences, University of São Paulo, São Paulo, Brazil; ^3^Laboratory of Protein Structure and Evolution, Department of Microbiology, Institute of Biomedical Sciences, University of São Paulo, São Paulo, Brazil

**Keywords:** *Mycobacterium bovis*, genome, comparative genomics, *Mycobacterium tuberculosis* complex, tuberculosis

## Abstract

*Mycobacterium bovis* causes bovine tuberculosis and is the main organism responsible for zoonotic tuberculosis in humans. We performed the sequencing, assembly and annotation of a Brazilian strain of *M. bovis* named SP38, and performed comparative genomics of *M. bovis* genomes deposited in GenBank. *M. bovis* SP38 has a traditional tuberculous mycobacterium genome of 4,347,648 bp, with 65.5% GC, and 4,216 genes. The majority of CDSs (2,805, 69.3%) have predictive function, while 1,206 (30.07%) are hypothetical. For comparative analysis, 31 *M. bovis*, 32 *M. bovis* BCG, and 23 *Mycobacterium tuberculosis* genomes available in GenBank were selected. *M. bovis* RDs (regions of difference) and Clonal Complexes (CC) were identified *in silico*. Genome dynamics of bacterial groups were analyzed by gene orthology and polymorphic sites identification. *M. bovis* polymorphic sites were used to construct a phylogenetic tree. Our RD analyses resulted in the exclusion of three genomes, mistakenly annotated as virulent *M. bovis*. *M. bovis* SP38 along with strain 35 represent the first report of CC European 2 in Brazil, whereas two other *M. bovis* strains failed to be classified within current CC. Results of *M. bovis* orthologous genes analysis suggest a process of genome remodeling through genomic decay and gene duplication. Quantification, pairwise comparisons and distribution analyses of polymorphic sites demonstrate greater genetic variability of *M. tuberculosis* when compared to *M. bovis* and *M. bovis* BCG (*p* ≤ 0.05), indicating that currently defined *M. tuberculosis* lineages are more genetically diverse than *M. bovis* CC and animal-adapted MTC (*M. tuberculosis* Complex) species. As expected, polymorphic sites annotation shows that *M. bovis* BCG are subjected to different evolutionary pressures when compared to virulent mycobacteria. Lastly, *M. bovis* phylogeny indicates that polymorphic sites may be used as markers of *M. bovis* lineages in association with CC. Our findings highlight the need to better understand host-pathogen co-evolution in genetically homogeneous and/or diverse host populations, considering the fact that *M. bovis* has a broader host range when compared to *M. tuberculosis*. Also, the identification of *M. bovis* genomes not classified within CC indicates that the diversity of *M. bovis* lineages may be larger than previously thought or that current classification should be reviewed.

## Introduction

Tuberculosis is an infectious disease that affects human beings and animals, and is caused by members of the *Mycobacterium tuberculosis* Complex (MTC). The MTC is composed of the human-adapted pathogens *M. tuberculosis* lineages 1 to 4 and *Mycobacterium africanum* lineages 5 and 6, and the animal-adapted species, *Mycobacterium bovis, Mycobacterium caprae, Mycobacterium pinnipedii, Mycobacterium microti, Mycobacterium orygis, Mycobacterium suricattae, Mycobacterium mungi*, “dassie bacillus” and “chimpanzee bacillus” (Coscolla and Gagneux, 2014; Rodriguez-Campos et al., 2014). These microorganisms have great genomic similarity (>99% of nucleotide identity in alignable regions) and have clonally evolved from a *M. tuberculosis*-like ancestor (Smith et al., 2009; Galagan, 2014). Despite their great genetic relatedness, MTC members are categorized into different species due to species-specific genotypic variations, such as single nucleotide polymorphisms (SNPs) and deletion of regions of difference (RDs), and phenotypic variations related to host adaptability and virulence (Brosch et al., 2002; Galagan, 2014; Rodriguez-Campos et al., 2014).

Nearly 10 million people are affected by tuberculosis worldwide every year. Although the majority of these cases are caused by *M. tuberculosis*, there is an underestimated proportion of zoonotic cases caused by *M. bovis*, the pathogen of bovine tuberculosis (Olea-Popelka et al., 2017). The burden of zoonotic tuberculosis is possibly a reflection of the nations' socioeconomic development and their ability to control the disease in cattle and pasteurize the milk. Unfortunately, bovine tuberculosis is still geographically widespread, especially in developing nations and countries with hard-to-control wildlife reservoirs, and causes significant economic losses for livestock producers (El-Sayed et al., 2016). In Brazil, the epidemiological situation of bovine tuberculosis is well-characterized in 75% of the cattle population; transversal studies showed prevalence of infected herds varying from 0.36 to 9.0% in different States. The disease is more prevalent in high-production dairy farms and the major risk factor for infection is the introduction of animals without tuberculin testing (Dias et al., 2016; Ferreira Neto et al., 2016; Ribeiro et al., 2016). Despite the fact that Brazil is the second world largest producer of bovine meat, little is known about *M. bovis* strains causing disease in the country, especially regarding their transmission capacity, virulence and genetic characteristics. Although few studies have shown variable virulence and transmissibility capabilities among different *M. bovis* strains, these have rarely included genomic analyses of these isolates (Aguilar León et al., 2009; Blanco et al., 2009; Meikle et al., 2011; Andrade et al., 2012; Wright et al., 2013; Garbaccio et al., 2014). Thus, in contrast to *M. tuberculosis*, the virulence and genomic characteristics of different *M. bovis* lineages are largely unexplored.

Comparative genomics studies among MTC members have been mostly performed aiming to understand *M. tuberculosis* features (Kato-Maeda et al., 2001; Ilina et al., 2013; Coscolla and Gagneux, 2014; Liu et al., 2014; Phelan et al., 2016). *M. bovis* genomes are generally included only for isolated comparison (Cole, 2002; Garcia Pelayo et al., 2009; Joshi et al., 2012; McGuire et al., 2012; Rue-Albrecht et al., 2014; de la Fuente et al., 2015; Periwal et al., 2015). Intra-species comparative genomics studies of *M. bovis* are related to epidemiologic investigations of outbreaks of animal tuberculosis using genome sequencing reads to detect SNPs and infer transmission networks (Biek et al., 2012; Glaser et al., 2016; Crispell et al., 2017). Only two studies using complete and draft genomes of *M. bovis* strains have been published. The first performed sequencing of three *M. bovis* and one *M. caprae* genomes to correlate genomic characteristics with virulence phenotypes (e.g., lesion scores and hosts) (de la Fuente et al., 2015). The second evaluated the phylogenetic relatedness of 38 *M. bovis* genomes, identifying recombination sites, and suggesting that some North-American lineages of *M. bovis* evolved faster than others, which might be related to bacterial maintenance in different hosts causing new outbreaks in the USA (Patané et al., 2017). Thus, a more comprehensive comparative genomic study of *M. bovis* strains using complete and draft genomes is still lacking and should provide important data to understand the biology of this pathogen, especially when compared to *M. tuberculosis*. Thus, the aims of the present study were to sequence, assemble, close, and annotate the genome of a Brazilian strain of *M. bovis* and perform a comparative genomic analysis with other virulent *M. bovis, M. bovis* BCG and *M. tuberculosis* genomes available in a public database.

## Materials and methods

### Isolation of *M. bovis* strain SP38 and DNA extraction

The Laboratory of Bacterial Zoonosis of the College of Veterinary Medicine, University of São Paulo, Brazil has been isolating *M. bovis* strains from all over the country since the 1990's. From this bacterial collection, a *M. bovis* strain was randomly selected to be sequenced in this study and called SP38. This strain was originally isolated from a granulomatous lesion collected from a bovine in a slaughterhouse located in the State of São Paulo, Brazil in 2010 (Morato et al., 2016). The lesion was cultured in Stonebrink medium as described previously (Ikuta et al., 2016), following standard procedures (Centro Panamericano de Zoonosis, 1985), and the isolate was stored at −20°C with no further passages. For the purpose of extracting DNA for this study, this isolate was reactivated in Stonebrink medium. After incubation, a single colony was selected and grown in fresh medium. When growth was detected, a sterile loop was used to collect bacterial mass and DNA was extracted as previously described (van Soolingen et al., 1994; Bemer-Melchior and Drugeon, 1999). All procedures were performed in a Biosafety Level 3+ Laboratory (BSL-3+ Prof. Dr. Klaus Eberhard Stewien) located at the Department of Microbiology, Institute of Biomedical Sciences, University of São Paulo, Brazil. Tubes containing DNA were properly disinfected, removed from BSL3+ and stored at −20°C until further analysis.

### DNA sequencing, genome assembly, and annotation

Extracted DNA was sent to the Genomics Core Facility of Purdue University, USA for sequencing. Briefly, the quality and concentration of the DNA were measure by Nanodrop 2000C (Thermo Fischer Scientific, Massachusetts, USA) and 0.8% agarose gel using a mass ladder. DNA was sheared into 800 bp (base pairs) fragments using the Hydroshear DNA Shearing Device (Genomic Solutions, Michigan, USA) to create fragments for paired end libraries. Sheared DNA was analyzed using a DNA High Sensitivity Chip in an Agilent 2100 Bioanalyzer (Agilent Technologies, California, USA), resulting in average fragment size of 804 bp. A paired-end genomic library was then constructed using TruSeq DNA PCR-free sample preparation kit (Illumina, California, USA) and 20% of a lane (Illumina v3 chemistry) of Illumina HiSeq2500 was used to sequence it.

*De novo* assembly of the sequenced reads was performed using ABySS software (Simpson et al., 2009) and CLC Genomics Workbench 8 (QIAGEN, Venlo, Holland). Additionally, reads were mapped to a reference genome (*M. bovis* strain AF2122/97—NC_002945.3) with CLC Genomics Workbench 8 (QIAGEN). ABySS generated contigs were ordered with Projector 2 (van Hijum et al., 2005) using the same reference genome. Contigs showing clear overlaps based on Smith-Waterman alignment, without repetitive sequences, were manually joined. Remaining gaps were closed using complementary information from CLC Genomic Workbench's *de novo* and reference mapping assemblies and/or using PCR followed by Sanger sequencing. Gene prediction and annotation was performed by NCBI Prokaryotic Genome Annotation Pipeline (PGAP).

### Comparative genomic analyses

As of 2016, 31 virulent *M. bovis* complete or draft genomes were available in GenBank and were selected for this study. These genomes were subjected to MTC species confirmation by manually checking the RD patterns *in silico* (presence or absence of RD1, RD4, RD9, RD12, RD1mic, and RD2seal) (Warren et al., 2006). Following this evaluation, 28 genomes of *M. bovis* were used in this study, which include the *M. bovis* strain SP38 and 3 other complete genomes and 24 drafts. These were from *M. bovis* isolated from cattle, wild boar, sheep and chimpanzee, in Brazil (10/28), Argentina (7/28), Spain (3/28), South Korea (3/28), United Kingdom (1/28), China (1/28), Uruguay (1/28), France (1/28), and Uganda (1/28) (Supplementary Table 1).

Also as of 2016, 37 genomes of *M. bovis* BCG were available in GenBank. After excluding drafts with high number of contigs (>321), 32 *M. bovis* BCG genomes (11 complete and 21 drafts) were included in this study (Supplementary Table 2). Additionally, 23 *M. tuberculosis* complete genomes were selected, also from GenBank, representing lineages 1 (Philippines and Indian Ocean), 2 (East Asia), 3 (India and East Africa), and 4 (Europe, America, Africa), and antibiotic sensitive and resistant strains (Supplementary Table 3).

For the phylogenomic analysis, the following MTC genomes were included (in addition to all 28 *M. bovis* and *M. tuberculosis* H37Rv): *M. africanum* GM041182, *M. africanum* MAL010070, *M. microti* 12, *M. caprae* MB2, *M. orygis* 112400015, *M. mungi* BM22813, and *M. suricattae* (Supplementary Table 4). All genomes are available in GenBank with the exception of *M. suricattae*. The reads of *M. suricattae* were obtained from ENA (European Nucleotide Archive).

### *In silico* spoligotyping of *M. bovis* genomes

For the spoligotype identification, the reads of *M. bovis* strain SP38 were analyzed in SpolPred (Coll et al., 2012), and the complete and draft genomes of *M. bovis* were investigated using SpoTyping (Xia et al., 2016). The resulting patterns were submitted to the *M. bovis* Spoligotype Database (www.mbovis.org) for spoligotype pattern identification.

### Clonal complexes of *M. bovis* genomes

The four Clonal Complexes (Müller et al., 2009; Berg et al., 2011; Smith et al., 2011; Rodriguez-Campos et al., 2012) were investigated among all 28 *M. bovis* genomes. *In silico* evaluation consisted in the evaluation of: RDAf1 presence or absence and deletion of spacer 30 for African 1 using three previously described primers to localize the sequences (Müller et al., 2009); RDAf2 presence or absence and deletion of spacers 3–7 for African 2 using three previously described primers to localize the sequences (Berg et al., 2011); RDEu1 presence or absence and deletion of spoligotype spacer 11 for Clonal Complex European 1 using a pair of previously described primers to localize the sequences (Smith et al., 2011) (Supplementary Table 5); SNP detection in the gene *gua*A at 3,765,573 position according to the reference genome (*M. bovis* strain AF2122/97) and deletion of spoligotype spacer 21 for European 2 (Rodriguez-Campos et al., 2012).

### Paralogous gene families

Paralogous gene families (PGF) of *M. bovis* strain SP38 were identified using BLASTClust, available online in the MPI bioinformatics Toolkit (Alva et al., 2016). Parameters of 70% coverage and 30% of identity were used. PGF were separated into functional categories according to COG (Cluster of Orthologous Group) (Tatusov et al., 2000).

### Gene orthology and synteny

For the identification of the pangenome, core and accessory genomes of *M. bovis*, all selected *M. bovis* genomes files in “.gbk” format were uploaded in KBase platform (Arkin et al., 2016). Pangenome analysis was performed with OrthoMCL (Li et al., 2003), selecting “Build Pangenome with OrthoMCL.” All CDSs (coding DNA sequences) were categorized according to COGs. When necessary (i.e., proteins identified as singletons or grouped as species or strain-specific orthologous clusters), individual protein sequences were analyzed using BLASTp and/or tBLATn (Altschul et al., 1990) against *M. bovis* and *M. tuberculosis* genomes.

For the identification of groups of orthologous proteins among virulent *M. bovis, M. bovis* BCG, and *M. tuberculosis*, all genomes were analyzed using Sybil, a comparative genomics platform provided by the Institute for Genome Sciences, University of Maryland, USA. Sybil and associated algorithms identified protein clusters using reciprocal best BLAST match corrected for paralogs as previously described (Crabtree et al., 2007; Riley et al., 2012). An all-vs-all BLASTp search identified pairs of best-hit in the genomes (*e*-value of 1e-05, 80% identity, 70% coverage). Paralogous genes were then clustered using Jaccard similarity coefficient, with cutoff of 0.6, for each protein. The resulting paralogous proteins and singletons were used to identify orthologous proteins among the genomes (Riley et al., 2012).

Synteny maps were also constructed using Sybil. These maps are characterized by a linear genome alignment arranged by CDSs in vertical bars, and are based on the orthologs identification procedure described above. Synteny plots are built by coloring CDSs of a selected reference genome in a gradient from yellow to blue (left to right). If the query genome shares an ortholog cluster with the reference genome, this cluster is indicated above the sequence using the color that corresponds to the query CDS position in its reference genome. This plot can provide information about gene order conservation, regions without orthology, and rearrangements among genomes (Riley et al., 2012). For this study, only complete genomes were analyzed in synteny maps: four genomes of virulent *M. bovis*, one of *M. bovis* BCG (strain Pasteur) and one of *M. tuberculosis* (strain H37Rv).

### Polymorphic sites

Genomes were clustered into three groups: virulent *M. bovis, M. bovis* BCG, and *M. tuberculosis*. Polymorphic sites were detected in each group with kSNP3 (Gardner et al., 2015). Detected mutations were annotated and identified as synonymous or non-synonymous. For the annotation, complete genomes of each group (four complete genomes of *M. bovis*, 11 of *M. bovis* BCG, and 23 of *M. tuberculosis*) were selected as references. Genes with polymorphic sites were then categorized according to COGs (Tatusov et al., 2000).

To determine which mycobacterium species group has the greatest number of polymorphic sites, pairwise analyses were performed in an all-against-all fashion for each group. Results were statistically compiled in GraphPad Prism 6 (GraphPad Software Inc, La Jolla, California, USA), where the three groups were compared using the non-parametric Kruskal–Wallis test, followed by Dunn test to detect differences between two groups. Results were considered statistically significant when *p* ≤ 0.05.

The distribution of core polymorphic sites according to the number of sequenced MTC genomes was also analyzed. For this analyses, the genomes were clustered into four groups: one group containing all selected *M. tuberculosis* genomes; one group containing animal-adapted MTC species (28 genomes of *M. bovis*, and one of each of *M. caprae, M. microti, M. mungi, M. suricattae*, and *M. orygis*); one group with all selected *M. bovis* genomes; and a group with all MTC genomes (all genomes described above and two genomes of *M. africanum* lineages 5 and 6). The convergence of the observed number of segregating sites using subsets of these groups was determined. Briefly, for a given number of genomes, a new multiple sequence alignment of the core polymorphic sites was generated by randomly selecting the same number of rows from the alignment of all core polymorphic sites generated by kSNP3 for each complete dataset. Every combination of genomes was sampled only once (i.e., without replacement) and the number of samples was limited to a maximum of 10,000 samples. The number of segregating sites was then calculated for each resampled alignment by counting all columns that contained at least one sequence with a non-conserved, i.e., divergent, nucleotide.

### Phylogenomic analysis

A phylogenetic tree based on core polymorphic sites was built using all selected *M. bovis* genomes, in addition to one of each MTC species. Polymorphic sites identification was performed with kSNP3, and the file “SNPs_all_matrix.fasta” was selected to construct a multiple sequence alignment in MEGA 7 (Kumar et al., 2016), using MUSCLE algorithm (Edgar, 2004). The resulting alignment was used to construct phylogenetic trees using Neighbor-Joining (NJ) and Maximum-Likelihood (ML) algorithms with bootstrap of 1,000 replications and Jukes Cantor substitution model.

## Results and discussion

### Genome of *M. bovis* strain SP38: a traditional tuberculous mycobacterium

A total of 29,651,856 reads containing 2,858,321,177 bases resulted from *M. bovis* SP38 genome sequencing (approximate coverage of 657x). First-pass assembly using filtered reads (5,930,382—with 571,709,745 bases) in ABySS software resulted in 51 contigs (4,373,651 bases), with a total of 68 gaps. Following contig ordering with Projector 2 and manual check for overlaps, most gaps were closed. Five remaining gaps were resolved using additional assemblies (*de novo* and “map reads to a reference genome” with CLC Genomics Workbench) and PCR followed by Sanger sequencing. Considering that there are no optimal assembly programs (Wences et al., 2015) and mycobacteria genomes are difficult to close due to repeat regions and high GC content (Kumar and Kaur, 2014), the combination of different assembly algorithms allowed us to obtain a complete genome without gaps.

A singular, circular, and complete genome of *M. bovis* strain SP38 with 4,347,648 bp was obtained, and submitted to GenBank to update the previous draft genome (Guimaraes et al., 2015) (updated accession number: NZ_CP015773.2). The PGAP annotation revealed a traditional tuberculous mycobacterium genome, with high GC content (65.6%) and 4,216 genes, including 154 pseudogenes, 3 rRNA genes (ribosomal RNA), 45 tRNA (transfer RNA), 2 ncRNA (non-coding RNA), 1 tmRNA (transfer-messenger RNA), and 4,011 CDSs. Among the CDSs, most (2,805/4,011; 69.93%) have predicted function, and 1,206 (30.07%) encode for hypothetical proteins. A total of 307 PGF (with 2 to 100 members each), representing 1,049/4,011 (26.79%) CDSs were also found. A great number (318/1,049; 30.31%) of these families encode for hypothetical proteins and/or PE/PPE proteins (proline-glutamic and proline-proline-glutamic, respectively). Similar to *M. tuberculosis* (Voskuil et al., 2004), 3.54% of the CDSs (142/4,011) in *M. bovis* strain SP38 are annotated as PE/PPE proteins. It is believed that these PGFs are important virulence factors, providing immune system evasion through genetic variation (Fishbein et al., 2015; Phelan et al., 2016). Finally, the spoligotype of *M. bovis* strain SP38 was identified as SB0121, the most prevalent spoligotype in Brazil (Rocha, 2013; Carvalho et al., 2016).

### Not all genomes available in genbank are virulent *M. bovis*

Three out of the 31 genomes identified in GenBank as *M. bovis* (3/31) did not have the RD pattern that is characteristic of this species. Accordingly, *M. bovis* strain ATCC BAA-935, demonstrated a RD pattern consistent with *M. bovis* BCG; the human isolate from Uganda, *M. bovis* strain B2 7505 (Wanzala et al., 2015), revealed *M. tuberculosis* RD patterns; and *M. bovis* strain MAL010093, also isolated from a human patient in Africa, was characterized as *M. africanum* (Supplementary Table 6). Although *M. bovis* ATCC BAA-935 is labeled in GenBank as AF2122/97, the original genome of strain AF2122/97 (NC_002945.4) has RD patterns consistent with virulent *M. bovis*. Thus, from the 31 selected genomes, 28 were confirmed as virulent *M. bovis* and used in further analysis. These findings warrant caution in species confirmation prior to depositing genome sequences in public databases. In order to avoid errors in comparative genomics analyses, it is essential that bacterial genomes are correctly classified. MTC species can be confirmed prior to genome sequencing through molecular diagnostic (Warren et al., 2006), or after acquiring genome reads using the software RD-analyzer (Faksri et al., 2016). This likely mistaken species definition led Patané et al. (2017) to suggest that *M. bovis* strain ATCC BAA-935, identified as BCG, was a virulent *M. bovis* with significant genetic recombination areas and large sequence polymorphisms (LSP).

### The most common spoligotype among genbank-deposited *M. bovis* genomes is SB0140

The majority (19/28, 67.86%) of the 28 *M. bovis* genomes available in GenBank have the spoligotype SB0140 (Supplementary Table 7). Spoligotype patterns from five *M. bovis* strains failed to be identified using the proposed software. Fortunately, the spoligotype classification of two of these strains (*M. bovis* strains MB1 and MB3) were described in a previous study (de la Fuente et al., 2015). The remaining three (*M. bovis* strains B_322, Bz 31150, and 30) were listed as unknown patterns.

Despite the fact that spoligotype SB0121 is considered the most prevalent in Brazil and in the Iberian Peninsula (Rodriguez-Campos et al., 2013; Zumárraga et al., 2013; Carvalho et al., 2016), only one Brazilian strain (*M. bovis* strain SP38) (1/10) analyzed herein demonstrated this pattern. The other national strains (9/10) were identified as SB0140, which is associated with the United Kingdom and is described as the most prevalent in Argentina (Zumárraga et al., 2013; Garbaccio et al., 2014) due to the importation of British cattle in that country (Zumárraga et al., 2013). In this context, all (7/7) Argentinian strains also demonstrated this pattern. The proximity of Brazil to Argentina and the livestock trade between these countries may explain why SB0140 has high frequency among Brazilian genomes available in GenBank. These results are obviously not representative of an epidemiological survey, but can be taken into consideration in further phylogenomic analysis, as described below.

### Clonal complex european 2 is found in brazil

The Clonal Complexes of *M. bovis* genomes are described below (Table 1 and Supplementary Table 8). Two genomes failed to be categorized using the current parameters: *M. bovis* strains D_10_02315 and MB3, both isolated from wild boars in France and Spain, respectively. Moreover, the Clonal Complex African 1 was not detected among the studied genomes, probably due to low geographical representativeness of the sample. The lack of Clonal Complex identification in two strains allow us to question if there are other lineages that have yet to be described, or if current complexes' patterns should be reviewed to include more genetically diverse strains.

**Table 1:** Identification of Clonal Complexes among the studied *Mycobacterium bovis* genomes.

**Genomes**	**Clonal complex**
*M. bovis* strain AF2122/97	European 1
*M. bovis* strain SP38	European 2
*M. bovis* strain 1595	European 1
*M. bovis* strain 30	European 1
*M. bovis* strain Bz 31150	African 2
*M. bovis* strain 04-303	European 1
*M. bovis* strain 09-1191	European 1
*M. bovis* strain 05-566	European 1
*M. bovis* strain 05-567	European 1
*M. bovis* strain 49-09	European 1
*M. bovis* strain 32-08	European 1
*M. bovis* strain 18-08C	European 1
*M. bovis* strain 35	European 2
*M. bovis* strain 08-08BF2	European 1
*M. bovis* strain 09-1193	European 1
*M. bovis* strain 534	European 1
*M. bovis* strain 0822-11	European 1
*M. bovis* strain 61-09	European 1
*M. bovis* strain 45-08b	European 1
*M. bovis* strain 09-1192	European 1
*M. bovis* strain 50	European 1
*M. bovis* strain W-1171	European 1
*M. bovis* strain MbURU-001	European 1
*M. bovis* strain MB4	European 2
*M. bovis* strain B-3222	European 1
*M. bovis* strain D_10_02315	?
*M. bovis* strain MB1	European 2
*M. bovis* strain MB3	?

*?, undetermined Clonal Complex*.

*Mycobacterium bovis* strain SP38, sequenced in this study, and the strain 35 of *M. bovis* constitute the first report of Clonal Complex European 2 in Brazil. Contrary to a previous study that reported that only 16% (6/36) of Brazilian *M. bovis* isolates are classified as Clonal Complex European 1 (Smith et al., 2011), this complex was identified as the most common in Brazilian *M. bovis* genomes available in GenBank (8/10). However, this data must be interpreted with caution, as the studied sample is not geographically representative. More studies will allow to confirm the distribution of Clonal Complexes in the Brazilian territory. Nevertheless, it is safe to conclude that Clonal Complexes European 1 and 2 can be found in Brazil.

### Genomic regions with altered synteny are identified among virulent *M. bovis* genomes

**Figure 1:**
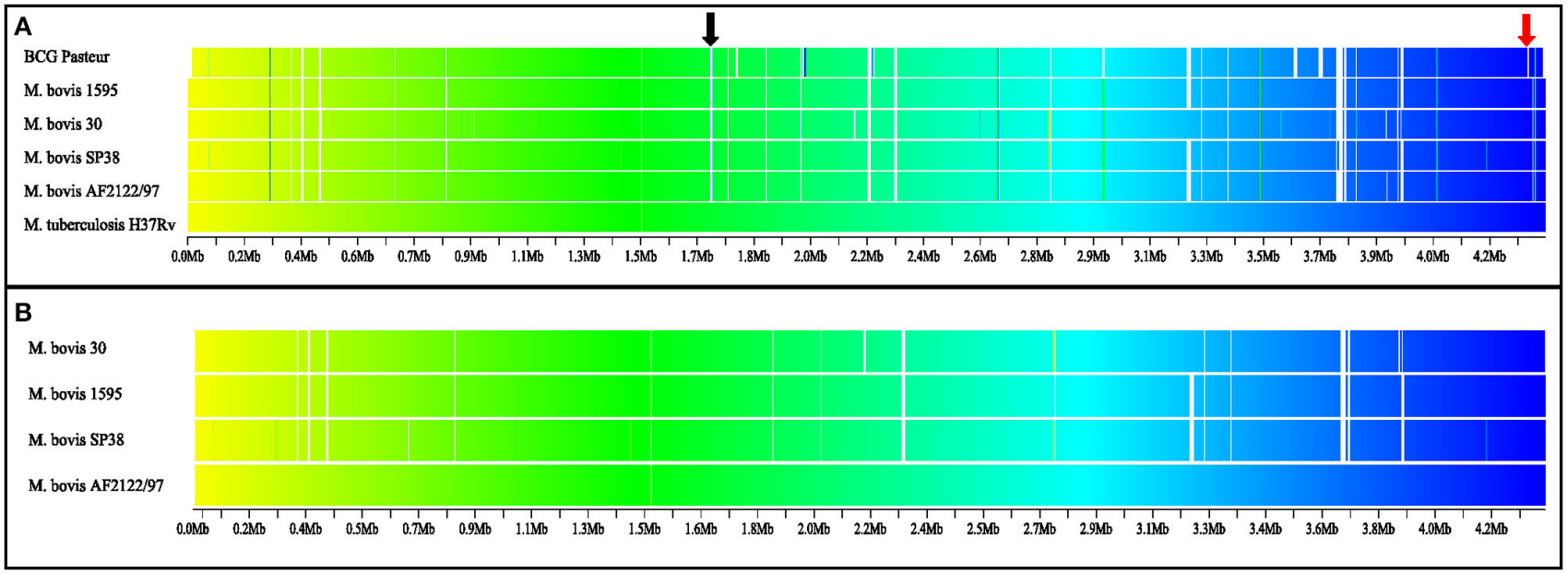
Synteny map of virulent *Mycobacterium bovis, M. bovis* BCG, and *M. tuberculosis* genomes. The gradient is based on color (yellow to blue; beginning to final), white indicates absence of orthologous genes. **(A)** Comparison among *M. bovis* and *M. bovis* BCG genomes with *M. tuberculosis* strain H37Rv as the reference genome. **(B)** Comparison among four *M. bovis* complete genomes, with *M. bovis* strain AF2122/97 as reference. BCG Pasteur: *M. bovis* BCG Pasteur 1173P2. RD4 (black arrow) absent in *M. bovis* and *M. bovis* BCG genomes, and RD1 (red arrow) absent in *M. bovis* BCG genome. RD, regions of difference.

As expected, synteny maps of complete *M. bovis* genomes and reference genomes of *M. tuberculosis* and *M. bovis* BCG showed great conservation of overall gene order (Figure 1). Nevertheless, few regions with loss of gene synteny were observed, mostly located between positions 2.2 and 4.3 Mb of the genomes. These may suggest an evolutionary process of gene loss through deletions (LSP, including known RDs highlighted in Figure 1) or pseudogenization (absence of orthologous genes due to early gene truncation) among virulent *M. bovis* species when compared to *M. tuberculosis* strain H37Rv (Figure 1A) or *M. bovis* AF2122/97 (Figure 1B). It was also possible to observe that *M. bovis* strain 30 has a synteny pattern slightly different when compared to other virulent *M. bovis*. More studies must be performed to individually identify these regions and their CDSs. It is possible that this information can be used to infer different lineages of *M. bovis*, as previously described in *M. tuberculosis*, in which different lineages have different RD/deletion profiles and specific gene mutations (Rodriguez-Campos et al., 2014).

### Pangenome analysis of *M. bovis* revealed an accessory genome with high number of CDSs with unknown function

*Mycobacterium bovis* pangenome is composed of 3,970 groups of orthologous CDSs and 1,329 singletons. Among the groups of orthologous CDSs, 3,149 are part of the core genome (i.e., CDSs presented in all 28 *M. bovis* genomes) and are functionally categorized in Table 2. Approximately half of these core groups are described as having unknown function (55.54%), represented mainly by hypothetical and PE/PPE family proteins. It is also important to highlight groups of CDSs involved in housekeeping functions, such as “transcription” (5.65%), and “lipid transport and metabolism” (5.14%), that contain primordial genes required for bacterium maintenance in the host (Raman et al., 2004) and to survival inside the macrophage (Fisher et al., 2002; Russell et al., 2010), respectively.

**Table 2:** *Mycobacterium bovis* orthologous CDSs of the core and accessory genomes categorized by COGs (Cluster of Orthologous Groups).

**COGs**	**Core**	**Accessory**
Cell cycle control, cell division, chromosome partitioning	20 (0.63%)	1 (0.12%)
Cell wall/membrane/envelope biogenesis	63 (2.00%)	9 (1.09%)
Cell motility	3 (0.09%)	1 (0.12%)
Post-translational modification, protein turnover, and chaperones	63 (2.00%)	2 (0.24%)
Signal transduction mechanisms	35 (1.11%)	7 (0.85%)
Intracellular trafficking, secretion, and vesicular transport	6 (0.19%)	0 (0.00%)
Defense mechanisms	49 (1.55%)	13 (1.58%)
Mobilome: transposons and prophages	24 (0.76%)	29 (3.53%)
RNA processing and modification	1 (0.03%)	0 (0.00%)
Translation, ribosomal structure and biogenesis	154 (4.89%)	14 (1.70%)
Transcription	178 (5.65%)	23 (2.80%)
Replication, recombination and repair	59 (1.87%)	8 (0.97%)
Energy production and conversion	95 (3.01%)	12 (1.46%)
Amino acid transport and metabolism	113 (3.58%)	15 (1.82%)
Nucleotide transport and metabolism	44 (1.39%)	3 (0.36%)
Carbohydrate transport and metabolism	59 (1.87%)	10 (1.21%)
Coenzyme transport and metabolism	92 (2.92%)	11 (1.34%)
Lipid transport and metabolism	162 (5.14%)	22 (2.68%)
Inorganic ion transport and metabolism	70 (2.22%)	12 (1.46%)
Secondary metabolites biosynthesis, transport, and catabolism	67 (2.12%)	12 (1.46%)
General function prediction only	152 (4.82%)	41 (5.00%)
Function unknown	1,749 (55.54%)	599 (72.96%)
Total of orthologous groups in core genome	3,149	821

*One protein can be categorized with more than one COG*.

The accessory genome of *M. bovis* [i.e., groups of CDSs present in at least two and up to 27 (~95%) *M. bovis* genomes] is composed of 821 groups of CDSs (Table 2). In contrast to the core groups, most of these are categorized as having unknown function (72.96%). Another important function that can be highlighted in addition to transcription and lipid metabolisms is the “mobilome: transposons and prophages” (3.53%), described as mobile genetic elements. These are represented by 29 groups of CDSs, present in 3 to 27 *M. bovis* genomes [the majority (28/29) are present in at least 20 genomes], and characterized as transposases of insertion sequences (IS), phiRV1 phage proteins and resolvases.

In *M. tuberculosis*, there are three main sources of repetitive DNA: duplicated genes and gene families, IS elements, and dispersed non-coding sequences (Cole, 1999). As described above, PE/PPE proteins are important duplicated genes in *M. bovis* strains. In addition, similarly to *M. tuberculosis* H37Rv, 10 IS families were found in *M. bovis* genomes (both in core and accessory genomes), named IS110, IS21, IS256, IS30, IS3, IS5, IS605, IS607, ISL3, and IS1535. The variable copy number of some of these IS in different *M. bovis* strains may indicate possible genetic markers for the epidemiological investigation of bovine tuberculosis.

Two prophage-like elements have been described in *M. tuberculosis* H37Rv genome, named phiRv1 and phiRv2. Their function is still uncertain, but appear to be related to host hypoxia (Fan et al., 2016). All strains of *M. tuberculosis* sequenced before September 2014 have either phiRv1 or phiRv2 (related to RD3 and RD11, respectively) (Fan et al., 2016). As expected (Brosch et al., 2002; Parsons et al., 2002), phiRv1 was found in all but two *M. bovis* genomes (*M. bovis* strains 0822-11 and Bz 31150) and phiRv2, which is generally not identified in this species (Behr et al., 1999; Parsons et al., 2002; Fan et al., 2016), has not been found in any of the analyzed genomes.

### *Mycobacterium bovis* singleton analysis demonstrates a process of genomic decay

OrthoMCL identified 1,329 proteins as being singletons, in other words, present only in one of the 28 *M. bovis* genomes. Of these, 824 (62%) were classified as hypothetical or PE/PPE family proteins and 505 (38%) have known function. Proteins with known function were analyzed using BLASTp and tBLASTn against MTC genomes. Twenty-four proteins were smaller than 30 aa, which is below the threshold allowed in OrthoMCL analysis, and 480/505 (95%) were found to be smaller in size when compared to other *M. bovis* proteins. In addition, there are on average 208 (range: 35 to 363) pseudogenes annotated per genome of *M. bovis*, in a total of 5,620 pseudogenes for 27 genomes (pseudogene information was not available for *M. bovis* strain 30). This corresponds to approximately 5% of the CDSs in a given *M. bovis* genome. As protein sequences of pseudogenes are not available in genome annotations, these were not included in the OrthoMCL analysis. Thus, these variable annotations may have interfered with the identification of singletons. It is also important to highlight that pseudogene annotation might be overestimated in draft genomes; CDSs located in contigs' extremities and without stop codon are annotated as pseudogenes.

From all these 1,329 proteins, only one protein was not identified in BLASTp or BLASTn analyses against *M. bovis* genomes: a membrane protein (341 aa), identified in the genome of *M. bovis* strain Bz 31150 (protein ID: WP_003899748.1), the sole African 2 representative in this study, and with 100% of identity to a probable conserved membrane protein of *M. tuberculosis* (protein ID: Rv3888c). Previous studies have shown that a 2.4 kb region involving membrane, ESX-2 type VII secretion system and ESAT-6 like proteins may or may not be present in *M. bovis* strains (Rauzier et al., 1999; Gey van Pittius et al., 2001). This region has been characterized as ESX-2 locus, one of the five ESAT-6 loci (Bitter et al., 2009; Gröschel et al., 2016). In contrast to many other ESX loci, ESX-2 distribution is restricted to certain mycobacterium species, suggesting that this is a system that arose more recently throughout evolution. Its function, however, remains unknown, as high-density transposon screens have shown that ESX-2 is not required for *in vitro* growth or virulence of *M. tuberculosis* in mice (Gröschel et al., 2016).

The above described characteristics are suggestive of a genomic decay process (gene loss and/or pseudogenization) of *M. bovis*, which is occurring intra-specifically and/or in comparison to other MTC members. The identified singletons are mostly a result of mutations that led to the premature truncation of their genes. Whether or not these are true pseudogenes warrants further investigation. In *M. tuberculosis* strains, pseudogenization has been linked to bacterial host specialization (Bäumler and Fang, 2013; Bolotin and Hershberg, 2015). It is believed that throughout evolution, environmental mycobacteria became specialized host pathogens by losing genes. The impact of this genomic remodeling through genomic decay and PE/PPE family protein duplication on MTC virulence and host adaptability is still unknown.

### Groups of orthologous proteins among *M. bovis, M. bovis* BCG, *M. tuberculosis*: there are no CDSs exclusively present in all *M. bovis* genomes

**Figure 2:**
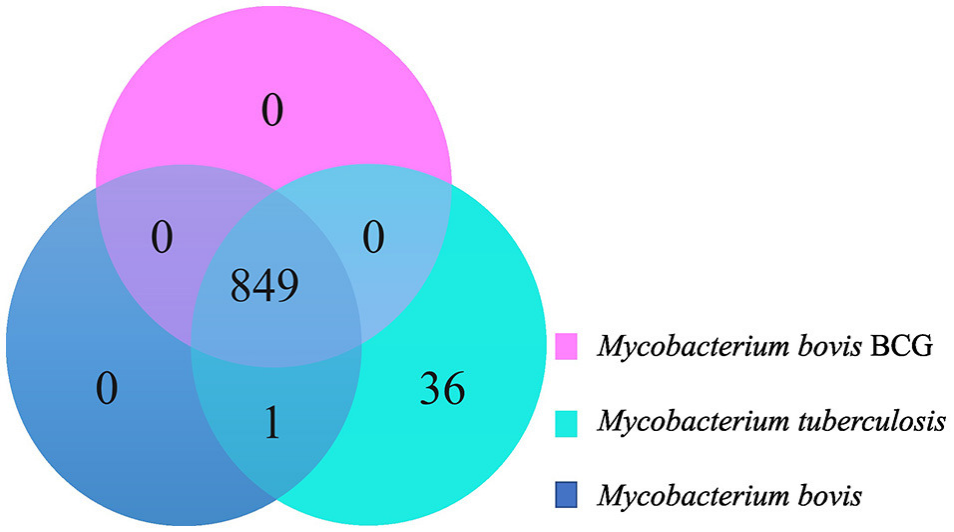
Venn Diagram demonstrating groups of orthologous genes shared among *Mycobacterium bovis* (dark blue), *M. bovis* BCG (pink), and *M. tuberculosis* (light blue).

The Venn diagram in figure 2 illustrates groups of orthologous proteins present in all the studied genomes of *M. bovis, M. bovis* BCG, and *M. tuberculosis*. There are no groups of orthologous proteins exclusively present in all 28 *M. bovis* genomes (Figure 2). The single group of orthologous proteins present exclusively among *M. bovis* and *M. tuberculosis* genomes and absent in *M. bovis* BCG is annotated as ESAT-6 proteins. These proteins are part of the RD1, an ~9 Kb attenuation-responsible region that codifies 9 CDSs of the ESX-1 locus (PE/PPE protein families and the virulence factor ESAT-6) (Mahairas et al., 1996; Brosch et al., 2002). Moreover, 36 groups of orthologous genes were exclusive of *M. tuberculosis* genomes (Figure 2, Supplementary Table 9). A great proportion of these groups (21/26; 58.33%) are related to RD4 and RD7. It is important to highlight the Mce (mammalian cell entry) 3 operon, which is related to the RD7 region and encodes invasin/adhesion proteins that have an important role in *M. tuberculosis* macrophage invasion and survival (Brosch et al., 2002, 2007; Haile et al., 2002; Ahmad et al., 2004). In addition, three groups identified as anti-anti-sigma factor RsfB, ESAT-6, and Cytochrome P450 (Rv3518) were found to be smaller in size (with or without enough amino acid sequence identity) in *M. bovis* genomes, precluding their clustering as orthologs, while other two (Pyridoxine/pyridoxamine 5′-phosphate oxidase and 6-phosphogluconate dehydrogenase Gnd1) were found to be smaller in size in *M. tuberculosis* genomes. These observations demonstrate once more the possibility of a genomic decay process or genome remodeling in *M. bovis* and *M. tuberculosis*.

### Selected *M. tuberculosis* strains showed a greater number of polymorphic sites when compared to virulent *M. bovis* and *M. bovis* BCG

**Figure 3:**
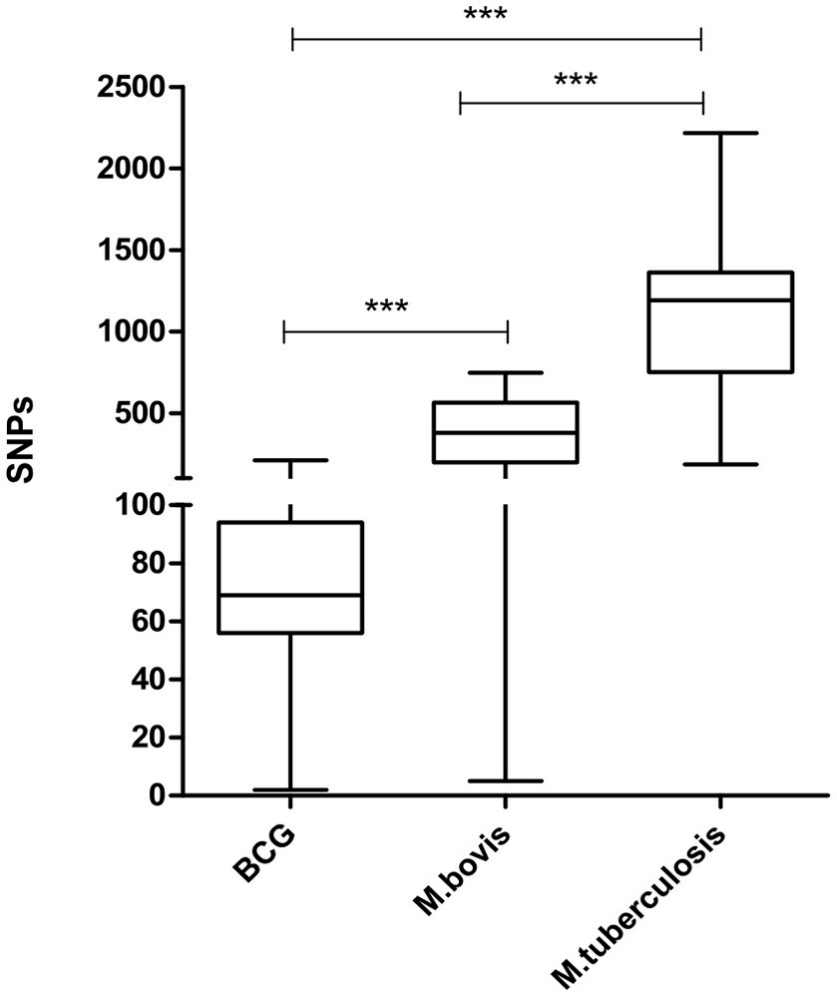
Distribution of whole genome pairwise comparisons of polymorphic sites. BCG: 32 genomes of *Mycobacterium bovis* BCG (mean: 76.83 SNPs; median: 69). *M. bovis:* 28 genomes of *Mycobacterium bovis* (mean: 381.8 SNPs; median: 378.5). *M. tuberculosis*: 23 genomes of *Mycobacterium tuberculosis* (mean: 1,192 SNPs; median: 1134.2). Bars indicate comparisons of the distribution of polymorphic sites among the three groups of mycobacterium species or between two groups of mycobacterium species (^***^p = 0.0001). Statistical analysis performed by Kruskal–Wallis, assuming *p* ≤ 0.05.

A total of 3,448 polymorphic sites were detected among virulent *M. bovis*, 1,088 among *M. bovis* BCG, and 8,335 among *M. tuberculosis* genomes. Pairwise analysis of each group indicates that *M. tuberculosis* has the highest number of polymorphic sites, followed by virulent *M. bovis*, and *M. bovis* BCG (*p* ≤ 0.05) (Figure 3). Even though *M. bovis* is considered a generalist species (i.e., it is able to infect multiple hosts), this mycobacterium demonstrated lower genetic variability when compared to *M. tuberculosis*. One possible explanation is that the *M. tuberculosis* sample analyzed herein is more geographically representative of the world strains when compared to *M. bovis*. However, it is also possible that *M. tuberculosis* strains present higher genetic variability due to prolonged co-evolution with human populations of various genetic backgrounds (Gagneux, 2012) or for being an evolutionarily older lineage of MTC.

As expected, the number of polymorphic sites was substantially lower in *M. bovis* BCG when compared to virulent *M. bovis* and *M. tuberculosis* (Figure 3). *M. bovis* BCG strains are solely subjected to artificial *in vitro* selection and originated from a single isolate. Nevertheless, the presence of variability in BCG strains should not be neglected, as they can reflect in different vaccine success rates (Garcia Pelayo et al., 2009).

### *M. tuberculosis* lineages are more genetically diverse than *M. bovis* clonal complexes

**Figure 4:**
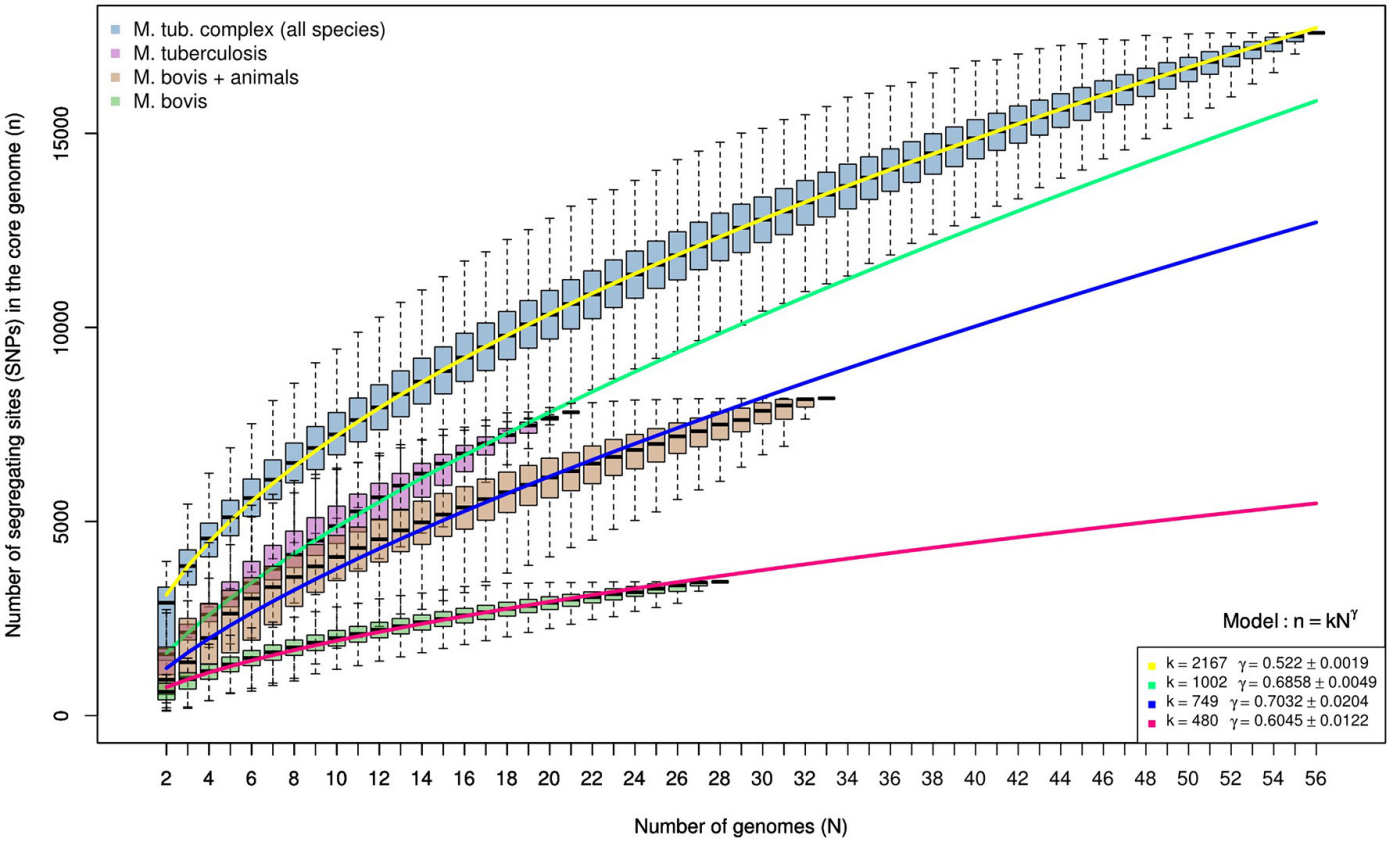
Spectrum for the number of segregating sites detected in random sub-samples of the core genome alignments produced by kSNP3. Each boxplot summarizes the distribution of segregating sites in up to 10,000 random samples of genomes with sizes indicated by the x-axis. The number of segregating sites appears in blue for the reduced MTC members' alignments, purple for *M. tuberculosis*, in brown for *M. bovis* and other animal adapted mycobacteria (*M. caprae, M. microti, M. mungi, M. suricattae, M. orygis*), and in green for *M. bovis*. Overlapping lines represent the best polynomial model (*n* = *kN*^γ^) fitted for each spectrum. Regardless of sample size and despite the inclusion of several more divergent animal-infecting *Mycobacterium* lineages to the *M. bovis* dataset, *M. tuberculosis* samples maintain consistently higher levels of genetic diversity.

Genomes of *M. tuberculosis* analyzed herein represent all four lineages of this species. The sole attempt to define *M. bovis* strains into lineages comes from the recent definition of Clonal Complexes. Selected *M. bovis* genomes represent 3 out of the 4 Clonal Complexes currently described (African 1 is the only missing complex). The evaluation of polymorphic sites as a function of the number of genomes being analyzed indicates that *M. tuberculosis* lineages are more diverse than *M. bovis* Clonal Complexes and possibly animal-adapted MTC species (*M. bovis, M. caprae, M. microti, M. mungi, M. suricattae*, and *M. orygis*) (Figure 4). This discrepancy as well as the definition of *M. bovis* lineages should be explored in future studies involving a more geographically representative *M. bovis* sample. And finally, it is clear that the diversity of MTC will continue to grow as additional genomes are sequenced, having a direct impact on the fight against these pathogens.

### Polymorphic sites annotation: *M. bovis* BCG is subjected to distinct evolutionary pressure when compared to virulent mycobacteria

Polymorphic sites annotation of *M. bovis, M. bovis* BCG, and *M. tuberculosis* are shown in Supplementary Tables 10–12. The proportion of synonymous (40.48–51.10%) vs. non-synonymous (48.90–59.52%) mutations was similar in all three groups. Of the 3,448 polymorphic sites identified in virulent *M. bovis* genomes, 2,804 (81.32%) are located within CDSs, 342 (9.92%) were not in annotated genome, 24 (0.70%) in non-protein coding regions, and 278 (8.06%) in unannotated regions. Similarly, of the 8,335 polymorphic sites identified in *M. tuberculosis* genomes, most of the mutations were located within CDSs (8,293, 99.50%). Only two (0.02%) polymorphic sites were not in annotated genomes, 10 (0.12%) in non-protein coding regions, and 30 (0.36%) in unannotated regions. On the other hand, from the 1,088 polymorphic sites described in *M. bovis* BCGs genomes, 757 (69.58%) of the mutations were identify within CDSs, 178 (16.36%) not in annotated genome, 52 (4.78%) in non-protein coding regions, and 101 (9.28%) in unannotated regions. The higher number of mutations in non-protein coding regions in *M. bovis* BCG when compared to virulent *M. bovis* and *M. tuberculosis* may be a result of the *in vitro* selective pressure that *M. bovis* BCG strains undergo. As intergenic regions may harbor regulatory regions, it is likely that mutations in such sites may lead to alterations in gene expression in these attenuated BCG strains.

The polymorphic site categorization according to COGs showed that *M. bovis* and *M. tuberculosis* have similar functional mutation patterns (Supplementary Tables 10, 11). In addition to CDSs categorized as having unknown and general functions, CDSs involved in “secondary metabolites biosynthesis, transport, and catabolism,” “lipid transport and metabolism,” “energy production and conversion,” and “amino acid transport and metabolism” represent a great proportion of the mutations. These shared patterns can be due to the intra-host natural selection pressure *M. tuberculosis* and *M. bovis* undergo in their lifecycle. Nevertheless, it is not possible to ignore that phenotype differences between both species may be due the presence of specific SNPs in certain genes (Filliol et al., 2006; Bigi et al., 2016, 2017).

In contrast to virulent mycobacteria, a significant proportion of polymorphic sites of *M. bovis* BCG were characterized as being in CDSs involved with “energy production and conversion,” followed by “cell cycle control, cell division, chromosome partitioning,” and “signal transduction mechanisms.” These characteristics reflect the *in vitro* selective pressure *M. bovis* BCG undergoes during its growth for vaccine production, contrasting with the intra-host environment encountered by virulent mycobacteria. Inside macrophages, virulent mycobacteria usually rely on lipids and cholesterol for growth (Pandey and Sassetti, 2008; Lee et al., 2013).

### Phylogenomics: *M. bovis* lineages form clades according to their clonal complexes

**Figure 5:**
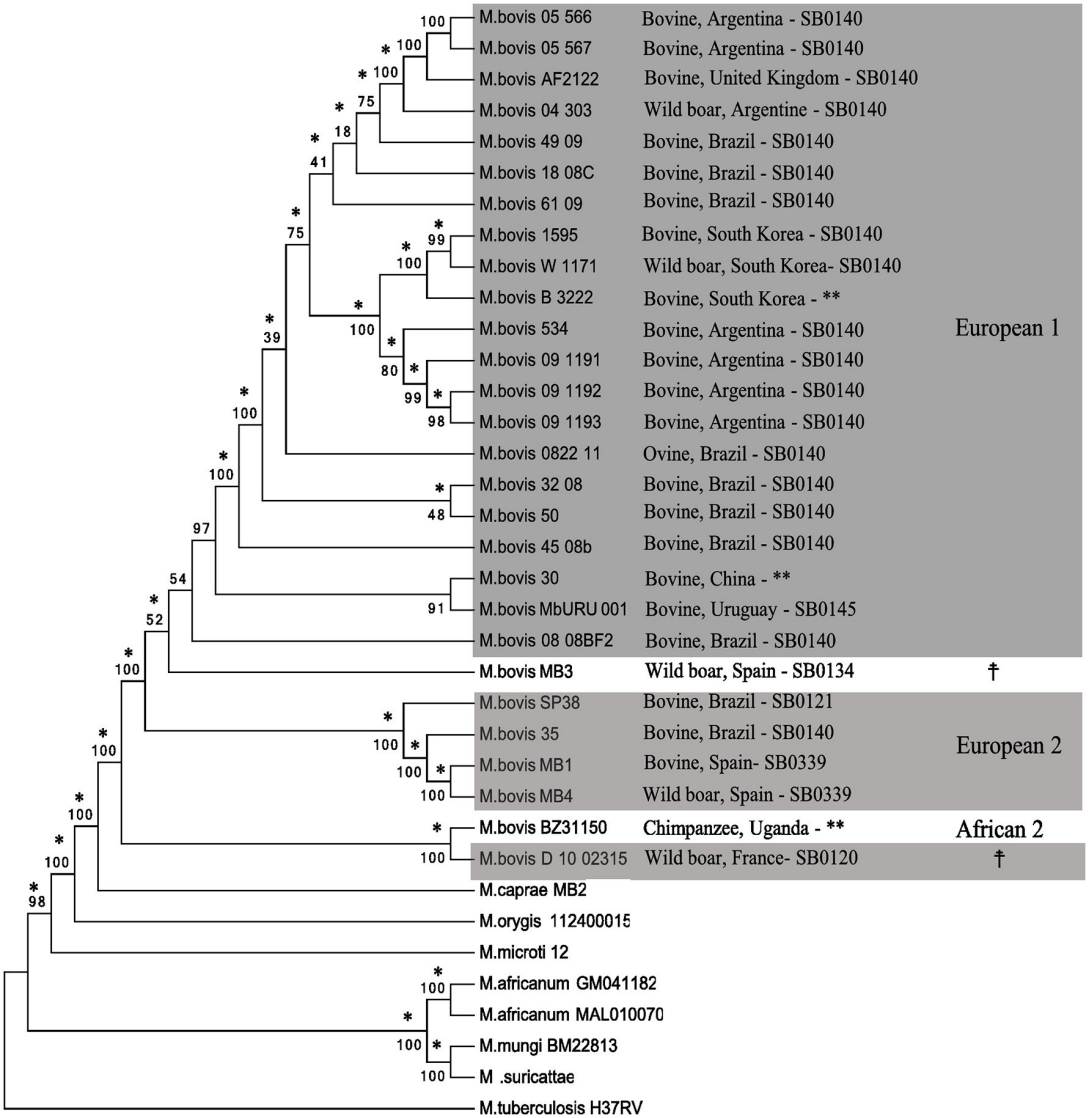
Phylogenetic tree based on SNPs (single nucleotide polymorphisms) of MTC (*Mycobacterium tuberculosis* Complex) members. The tree was generated from a multiple sequence alignment using Neighbor-Joining (shown) and Maximum Likelihood (not shown) algorithms with 1,000 bootstrap replicates. Mycobacterium tuberculosis H37Rv was used as an outgroup. *Mycobacterium bovis* genomes are identified by host species, country of isolation, SB number, and Clonal Complexes (gray boxes). Branches that agree with the Maximum-likelihood tree are indicated with an asterisk (^*^). ^**^Uncharacterized SB number. 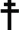
Undetermined Clonal Complex.

The phylogenetic tree based on polymorphic sites supports the classification of *M. bovis* into clades according to their Clonal Complexes, and not according to the host in which they were isolated from (Figure 5). The identification and analyses of SNPs in MTC genomes is an important tool used for their phylogenetic reconstruction and discrimination among different lineages, as described for *M. tuberculosis*, and as a marker to distinguish MTC species (Gutacker et al., 2002; Namouchi et al., 2012; Coll et al., 2014; Coscolla and Gagneux, 2014; Phelan et al., 2016). There are few studies regarding the comparative genomics of *M. bovis* (Garcia Pelayo et al., 2009; Joshi et al., 2012; de la Fuente et al., 2015; Patané et al., 2017) and, to our knowledge, this is the first study involving SNP phylogenomics of *M. bovis* genomes deposited in GenBank, complete or draft, in association with Clonal Complexes. Even though representatives of the Clonal Complex African 1 were not present in our sample, these results support the use of SNPs as a possible phylogenetic marker of *M. bovis* in association with Clonal Complexes, which can be explored to distinguish this species into different lineages.

### Final considerations

This is the first study to sequence and close a complete genome of a Brazilian strain of *M. bovis*. The *M. bovis* strain SP38 genome demonstrated traditional characteristics of a tuberculous mycobacterium genome, and was categorized as Clonal Complex European 2, the first report of this Clonal Complex in Brazil. Comparative genomics of *M. bovis* SP38 and other *M. bovis* genomes deposited in GenBank demonstrated an active process of intra-species genomic decay. The remodeling of mycobacterium genomes through gene loss (deletion or pseudogenization) and duplication (particularly of PE/PPE family genes) must be comparatively explored among MTC species regarding their impact on host adaption and virulence. Also, the identification of *M. bovis* genomes that fail to be classified within current Clonal Complexes indicates that the diversity of *M. bovis* may be larger than previously thought or that current classification needs to be reviewed. The detected smaller genetic diversity of *M. bovis* when compared to *M. tuberculosis* genomes contrasts with the generalist behavior of this species. In fact, *M. tuberculosis* lineages appeared more genetically diverse than animal-adapted MTC species. This finding highlights the need to better understand host-pathogen co-evolution in the context of genetically homogeneous and/or diverse host populations. And finally, the phylogeny based on SNPs can be used as a complementary tool for *M. bovis* Clonal Complex identification and explored as a gold-standard for lineages classification of *M. bovis* strains, as performed for *M. tuberculosis*.

## Authors contributions

CZ, PB, AdS, RdS, CI, NC and AG: conceived and designed experiments; CZ, RdS, NC and AG: analyzed the data; PB, JF, MH and AG: contributed reagents, materials, analysis tools; CZ, RdS and AG, wrote the paper; CZ, PB, AdS, RdS, CI, JF, NC, MH and AG: approved the paper.

### Conflict of interest statement

The authors declare that the research was conducted in the absence of any commercial or financial relationships that could be construed as a potential conflict of interest.
