# Supplementary material for: Complete Genome Sequencing of Mycobacterium bovis SP38 and Comparative Genomics of Mycobacterium bovis and M. tuberculosis Strains
Source: Front Microbiol. 2017 Dec 5;8:2389. doi: 10.3389/fmicb.2017.02389 (PMC5723337; doi:10.3389/fmicb.2017.02389)
Supplement: Supplementary file 1 [file Table1.DOCX]

Supplementary Material

# Complete genome sequencing and comparative genomics of *Mycobacterium bovis* SP38

Cristina Kraemer Zimpel, Paulo Eduardo Brandão, Antônio Francisco de Souza Filho, Robson Francisco de Souza, Cássia Yumi Ikuta, José Soares Ferreira Neto, Naila Cristina Soler Camargo, Marcos Bryan Heinemann, Ana Marcia Sá Guimarães^*^

***Correspondence:** Dr Ana Marcia Sa Guimaraes: anamarcia@usp.br

Supplementary Table 1. Selected *Mycobacterium bovis* genomes available in GenBank as of 2016.

| *M. bovis* genomes | Accession Number | Host | Country | Genomic feature |
| --- | --- | --- | --- | --- |
| AF2122/97 | NC_002945.3 | Bovine | United Kingdom | Complete |
| SP38 | NZ_CP015773.1 | Bovine | Brazil | Complete |
| 1595 | NZ_CP012095.1 | Bovine | South Korea | Complete |
| 30 | CP010332.1 | Bovine | China | Complete |
| Bz 31150 | NZ_JKAM00000000.1 | Chimpanzee | Uganda | Draft |
| 04-303 | NZ_AVSW00000000.1 | Wild boar | Argentina | Draft |
| 09-1191 | NZ_JPFP00000000.1 | Bovine | Argentina | Draft |
| 05-567 | NZ_JPFQ00000000.1 | Bovine | Argentina | Draft |
| 05-566 | NZ_JPFR00000000.1 | Bovine | Argentina | Draft |
| 49-09 | NZ_JQES00000000.1 | Bovine | Brazil | Draft |
| 32-08 | NZ_JQER00000000.1 | Bovine | Brazil | Draft |
| 18-08C | NZ_JQEQ00000000.1 | Bovine | Brazil | Draft |
| 35 | NZ_JQEV00000000.1 | Bovine | Brazil | Draft |
| 08-08BF2 | NZ_JQET00000000.1 | Bovine | Brazil | Draft |
| 09-1193 | NZ_JQEN00000000.1 | Bovine | Argentina | Draft |
| 534 | NZ_JQEM00000000.1 | Bovine | Brazil | Draft |
| 0822-11 | NZ_JQEW00000000.1 | Ovine | Argentina | Draft |
| 61-09 | NZ_JQEX00000000.1 | Bovine | Brazil | Draft |
| 45-08B | NZ_JQEP00000000.1 | Bovine | Brazil | Draft |
| 09-1192 | NZ_JQEO00000000.1 | Bovine | Argentina | Draft |
| 50 | NZ_JQEU00000000.1 | Bovine | Brazil | Draft |
| W-1171 | NZ_JXTK00000000.1 | Wild boar | South Korea | Draft |
| MbURU-001 | NZ_LFGY00000000.1 | Bovine | Uruguay | Draft |
| MB4 | NZ_CDHE00000000.1 | Wild boar | Spain | Draft |
| B-3222 | NZ_LNOF00000000.1 | Bovine | South Korea | Draft |
| D-10-02315 | NZ_MINA00000000.1 | Wild boar | France | Draft |
| MB1 | NZ_CDHF00000000.1 | Bovine | Spain | Draft |
| MB3 | NZ_CDHH00000000.1 | Wild boar | Spain | Draft |
