# Supplementary material for: Complete Genome Sequencing of Mycobacterium bovis SP38 and Comparative Genomics of Mycobacterium bovis and M. tuberculosis Strains
Source: Front Microbiol. 2017 Dec 5;8:2389. doi: 10.3389/fmicb.2017.02389 (PMC5723337; doi:10.3389/fmicb.2017.02389)
Supplement: Supplementary file 2 [file Table2.DOCX]

Supplementary Table 2. Selected *Mycobacterium bovis* BCG genomes available in GenBank as of 2016.

| *M. bovis* BCG Genome | Accession Number | Genomic feature |
| --- | --- | --- |
| Pasteur 1173P2 | NC_008769.1/AM408590.1 | Complete |
| Tokyo 172 | NC_012207.1/AP010918.1 | Complete |
| ATCC 35743 | NZ_CP003494.1/CP003494.1 | Complete |
| Mexico | NC_016804.1/CP002095.1 | Complete |
| Korea 1168P | NC_020245.2/CP003900.2 | Complete |
| Moreau RDJ | NZ_AM412059.1/AM412059.2 | Complete |
| BCG_3281 | NZ_CP008744.1/CP008744.1 | Complete |
| Russia 368 | NZ_CP009243.1/CP009243.1 | Complete |
| BCG-1 (Russia) | NZ_CP013741.1/CP013741.1 | Complete |
| 26 | CP010331.1 | Complete |
| Tokyo 172 TRCS | NZ_CP014566.1/CP014566.1 | Complete |
| BCG-1 | NZ_CP011455.1/CP011455.1 | Draft |
| China | NZ_AEZE00000000.1 | Draft |
| ATCC 35733 | NZ_AEZF00000000.1 | Draft |
| ATCC 35740 | NZ_AEZG00000000.1 | Draft |
| Frappier | NZ_AKYQ00000000.1 | Draft |
| BCG_jnaf | NZ_JNAF00000000.1 | Draft |
| BCG_cys | NZ_CYST00000000.1 | Draft |
| Phipps | NZ_CUWN00000000.1 | Draft |
| Moreau | NZ_CUWK00000000.1 | Draft |
| Birkhaug | NZ_CUWE00000000.1 | Draft |
| Sweden | NZ_CUWP00000000.1 | Draft |
| Danish | NZ_CUWH00000000.1 | Draft |
| Pasteur | NZ_CUWL00000000.1 | Draft |
| Glaxo | NZ_CUWJ00000000.1 | Draft |
| Russia | NZ_CUWO00000000.1 | Draft |
| Prague | NZ_CUWM00000000.1 | Draft |
| China | NZ_CUWG00000000.1 | Draft |
| Connaught | NZ_CUWF00000000.1 | Draft |
| Tice | NZ_CUWQ00000000.1 | Draft |
| Japan | NZ_CUWR00000000.1 | Draft |
| Frappier | NZ_CUWI00000000.1 | Draft |
