# Supplementary material for: Complete Genome Sequencing of Mycobacterium bovis SP38 and Comparative Genomics of Mycobacterium bovis and M. tuberculosis Strains
Source: Front Microbiol. 2017 Dec 5;8:2389. doi: 10.3389/fmicb.2017.02389 (PMC5723337; doi:10.3389/fmicb.2017.02389)
Supplement: Supplementary file 3 [file Table3.DOCX]

Supplementary Table 3. Selected *Mycobacterium tuberculosis* genomes available in GenBank as of 2016.

| *M. tuberculosis* genomes | Accession Number | Country | Characteristics |
| --- | --- | --- | --- |
| H37Rv | NC_000962.3 | United Kingdom | Lineage 4 |
| CDC1551 | NC_002755.2/AE000516.2 | USA | Lineage 4 |
| F11 | NC_009565.1/CP000717.1 | South Africa | Drug Susceptible |
| KZN 1435 | NC_012943.1/CP001658.1 | South Africa | Multidrug Resistant |
| Haarlem | NC_022350.1/CP001664.1 | - | Multidrug Resistant |
| CCDC5180 | NC_017522.1/CP001642.1 | China | Multidrug Resistant |
| 7199-99 | NC_020089.1/HE663067.1 | Germany | - |
| Beijing/NITR203 | NC_021054.1/CP005082.1 | India | - |
| CCDC5079 | NC_021251.1/CP002884.1 | China | Drug Susceptible |
| EAI5 | NC_021740.1/CP006578.1 | India | - |
| KIT87190 | NZ_CP007809.1/CP007809.1 | South Korea | - |
| 96075 | NZ_CP009426.1/CP009426.1 | China | Lineage 2 |
| 96121 | NZ_CP009427.1/CP009427.1 | Filipinas | Lineage 1 |
| Kurono | NZ_AP014573.1/AP014573.1 | Japan | Lineage 4 |
| SCAID 187.0 | NZ_CP012506.2/CP012506.2 | Kazakhstan | Multidrug Resistant |
| 2242 | CP010335.1 | China | Lineage 2 |
| 2279 | CP010336.1 | China | Lineage 2 |
| 22115 | NZ_CP010337.1/CP010337.1 | China | Lineage 4 |
| 37004 | NZ_CP010338.1/CP010338.1 | China | Lineage 4 |
| 26105 | NZ_CP010340.1/CP010340.1 | China | Lineage 3 |
| Beijing-like | CP010873.1 | Colombia | - |
| ZMC13-264 | NZ_CP009100.1/CP009100.1 | China | Multidrug Resistant |
| ZMC13-88 | NZ_CP009101.1/CP009101.1 | China | Multidrug Resistant |
