# Supplementary material for: Complete Genome Sequencing of Mycobacterium bovis SP38 and Comparative Genomics of Mycobacterium bovis and M. tuberculosis Strains
Source: Front Microbiol. 2017 Dec 5;8:2389. doi: 10.3389/fmicb.2017.02389 (PMC5723337; doi:10.3389/fmicb.2017.02389)
Supplement: Supplementary file 4 [file Table4.DOCX]

Supplementary Table 4. Selected *Mycobacterium tuberculosis* complex genomes available in GenBank and ENA* (European Nucleotide Archives) as of 2016.

| Genomes | Accession Number | Genomic feature | Characteristic |
| --- | --- | --- | --- |
| *M. africanum* GM041182 | NC_015758.1/FR878060.1 | Complete | Lineage 6 |
| *M. africanum* MAL010070 | NZ_JLAZ00000000.1 | Draft | Lineage 5 |
| *M. microti* 12 | CP010333.1 | Draft | - |
| *M. caprae* MB2 | NZ_CDHG00000000.1 | Draft | - |
| *M. orygis* 112400015 | NZ_APKD00000000.1 | Draft | - |
| *M. mungi* BM22813 | NZ_LXTB00000000.1 | Draft | - |
| *M. suricattae* | ERX1047373* | Reads | - |
