# Supplementary material for: Complete Genome Sequencing of Mycobacterium bovis SP38 and Comparative Genomics of Mycobacterium bovis and M. tuberculosis Strains
Source: Front Microbiol. 2017 Dec 5;8:2389. doi: 10.3389/fmicb.2017.02389 (PMC5723337; doi:10.3389/fmicb.2017.02389)
Supplement: Supplementary file 5 [file Table5.DOCX]

Supplementary Table 5. Regions of Difference (RDs) evaluated in *Mycobacterium bovis* genomes for determination of the Clonal Complexes European 1, African 1 and 2.

| Marker | Profile | Clonal Complex |
| --- | --- | --- |
| RDAf1 | Intact - 350 bp; deletion - 531 bp | African 1 - deletion RDAf1 |
| RDAf2 | Intact - 451 bp; deletion - 711 bp | African 2 - deletion RDAf2 |
| RDEu1 | Intact - 1.207 bp; deletion - 400 bp | European 1 - deletion RDEu1 |

bp = base pairs
