# Supplementary material for: Complete Genome Sequencing of Mycobacterium bovis SP38 and Comparative Genomics of Mycobacterium bovis and M. tuberculosis Strains
Source: Front Microbiol. 2017 Dec 5;8:2389. doi: 10.3389/fmicb.2017.02389 (PMC5723337; doi:10.3389/fmicb.2017.02389)
Supplement: Supplementary file 6 [file Table6.DOCX]

Supplementary Table 6. *Mycobacterium bovis* genomes available in GenBank as of 2016 identified with non-*Mycobacterium bovis* RD (regions of difference) patterns.

| *M. bovis* genomes | Accession Number | RD | Corrected Genome |
| --- | --- | --- | --- |
| ATCC BAA-935 | NZ_CP009449.1/  [CP009449.1](https://www.ncbi.nlm.nih.gov/nuccore/CP009449.1) | RD1 absent (192 bp)  RD4 absent (267 bp)  RD9 absent (107 bp) | *M. bovis* BCG |
| B2 7505 | NZ_JKAL00000000.1 | RD1 presence (146 bp)  RD4 presence (172 bp)  RD9 presence (235 bp)  RD12 presence (369 bp) | *M. tuberculosis* |
| MAL_010093 | NZ_JLAP00000000.1 | RD1 presence (146 bp)  RD4 presence (172 bp)  RD9 absent (108 bp)  RD12 presence (369 bp)  1mic presence (195 bp)  2seal absent (no sequence) | *M. africanum* |

bp = base pairs
