# Supplementary material for: Complete Genome Sequencing of Mycobacterium bovis SP38 and Comparative Genomics of Mycobacterium bovis and M. tuberculosis Strains
Source: Front Microbiol. 2017 Dec 5;8:2389. doi: 10.3389/fmicb.2017.02389 (PMC5723337; doi:10.3389/fmicb.2017.02389)
Supplement: Supplementary file 7 [file Table7.DOCX]

Supplementary Table 7. Spoligotyping patterns among *Mycobacterium bovis* genomes deposited in GenBank as of 2016.

| ***M. bovis* genomes** | **Binnary pattern** | **OCTcode pattern** | **SB pattern** |
| --- | --- | --- | --- |
| AF2122/97 | 1101101000001110111111111111111111111100000 | 664073777777600 | SB0140 |
| SP38 | 1101111101111110111101111111111111111100000 | 676773677777600 | SB0121 |
| 1595 | 1101101000001110111111111111111111111100000 | 664073777777600 | SB0140 |
| 30 | 1101011101011110110111111110111111111100000 | 656573377377600 | ? |
| Bz 31150 | 0000000000000000000011111100000000000100000 | 000000176000200 | ? |
| 04-303 | 1101101000001110111111111111111111111100000 | 664073777777600 | SB0140 |
| 09-1191 | 1101101000001110111111111111111111111100000 | 664073777777600 | SB0140 |
| 05-566 | 1101101000001110111111111111111111111100000 | 664073777777600 | SB0140 |
| 05-567 | 1101101000001110111111111111111111111100000 | 664073777777600 | SB0140 |
| 49-09 | 1101101000001110111111111111111111111100000 | 664073777777600 | SB0140 |
| 32-08 | 1101101000001110111111111111111111111100000 | 664073777777600 | SB0140 |
| 18-08C | 1101101000001110111111111111111111111100000 | 664073777777600 | SB0140 |
| 35 | 1101101000001110111111111100001111111100000 | 664073776077600 | SB0140 |
| 08-08BF2 | 1101101000001110111111111111111111111100000 | 664073777777600 | SB0140 |
| 09-1193 | 1101101000001110111111111111111111111100000 | 664073777777600 | SB0140 |
| 534 | 1101101000001110111111111111111111111100000 | 664073777777600 | SB0140 |
| 0822-11 | 1101101000001110111111111111111111111100000 | 664073777777600 | SB0140 |
| 61-09 | 1101101000001110111111111111111111111100000 | 664073777777600 | SB0140 |
| 45-08b | 1101101000001110111111111111111111111100000 | 664073777777600 | SB0140 |
| 09-1192 | 1101101000001110111111111111111111111100000 | 664073777777600 | SB0140 |
| 50 | 1101101000001110111111111111111111111100000 | 664073777777600 | SB0140 |
| W-1171 | 1101101000001110111111111111111111111100000 | 664073777777600 | SB0140 |
| MbURU-001 | 1101000000000010111111111111111111111100000 | 640013777777600 | SB0145 |
| MB4 | 1101111101111110111101111000011111111100000 | 676773674177600 | SB0339 |
| B-3222 | 1101101000000000111111111111111111001100000 | 664003777774600 | ? |
| D_10_02315 | 1101111101111110111111111111111111111100000 | 676773777777600 | SB0120 |
| MB1 | 1101111000000000000000000000000000011100000 | 674000000001600 | SB0339* |
| MB3 | 1100000000000000011111111000000000001100000 | 600001774000600 | SB0134* |

? undetermined spoligotype pattern
