# Supplementary material for: Complete Genome Sequencing of Mycobacterium bovis SP38 and Comparative Genomics of Mycobacterium bovis and M. tuberculosis Strains
Source: Front Microbiol. 2017 Dec 5;8:2389. doi: 10.3389/fmicb.2017.02389 (PMC5723337; doi:10.3389/fmicb.2017.02389)
Supplement: Supplementary file 8 [file Table8.DOCX]

Supplementary Table 8. Clonal Complexes of *Mycobacterium bovis* genomes deposited in GenBank as of 2016.

| ***M. bovis* genomes** | **Profile** | **Clonal Complex** |
| --- | --- | --- |
| AF2122/97 | RDEu1 absent (401 bp) and absence of spacer 11 | European 1 |
| SP38 | SNP in guaA gene and absence of spacer 11, 21 and 30 | European 2 |
| 1595 | RDEu1 absent (401 bp) and absence of spacer 11 | European 1 |
| 30 | RDEu1 absent (401 bp) and absence of spacer 11 | European 1 |
| Bz 31150 | RDAf1 absent (711 bp) and absence of spacer 3 to 7, 11 and 30 | African 2 |
| 04-303 | RDEu1 absent (401 bp) and absence of spacer 11 | European 1 |
| 09-1191 | RDEu2 absent (401 bp) and absence of spacer 11 | European 1 |
| 05-566 | RDEu1 absent (401 bp) and absence of spacer 11 | European 1 |
| 05-567 | RDEu1 absent (401 bp) and absence of spacer 11 | European 1 |
| 49-09 | RDEu1 absent (401 bp) and absence of spacer 11 | European 1 |
| 32-08 | RDEu1 absent (401 bp) and absence of spacer 11 | European 1 |
| 18-08C | RDEu1 absent (401 bp) and absence of spacer 11 | European 1 |
| 35 | SNP in guaA gene and absence of spacer 11 | European 2 |
| 08-08BF2 | RDEu1 absent (401 bp) and absence of spacer 11 | European 1 |
| 09-1193 | RDEu1 absent (401 bp) and absence of spacer 11 | European 1 |
| 534 | RDEu1 absent (401 bp) and absence of spacer 11 | European 1 |
| 0822-11 | RDEu1 absent (401 bp) and absence of spacer 11 | European 1 |
| 61-09 | RDEu1 absent (401 bp) and absence of spacer 11 | European 1 |
| 45-08b | RDEu1 absent (401 bp) and absence of spacer 11 | European 1 |
| 09-1192 | RDEu1 absent (401 bp) and absence of spacer 11 | European 1 |
| 50 | RDEu1 absent (401 bp) and absence of spacer 11 | European 1 |
| W-1171 | RDEu1 absent (401 bp) and absence of spacer 11 | European 1 |
| MbURU-001 | RDEu1 absent (401 bp) and absence of spacer 11 | European 1 |
| MB4 | SNP in guaA gene and absence of spacer 21 | European 2 |
| B-3222 | RDEu1 absent (401 bp) and absence of spacer 11 | European 1 |
| D_10_02315 | RDEu1 intact (1.207 bp), RDAf1 intact (350 bp), RDAf2 intact (451 bp), lack of SNP in guaA gene, absence of spaceres 3 to 7, 11, 30 and presence of 21 | ? |
| MB1 | SNP in guaA gene and absence of spacer 11, 21 and 30 | European 2 |
| MB3 | RDEu1 intact (1.207 bp), RDAf1 intact (350 bp), RDAf2 intact (451 bp), lack of SNP in guaA gene | ? |

? undetermined Clonal Complex; bp = base pairs
