# Supplementary material for: Complete Genome Sequencing of Mycobacterium bovis SP38 and Comparative Genomics of Mycobacterium bovis and M. tuberculosis Strains
Source: Front Microbiol. 2017 Dec 5;8:2389. doi: 10.3389/fmicb.2017.02389 (PMC5723337; doi:10.3389/fmicb.2017.02389)
Supplement: Supplementary file 9 [file Table9.DOCX]

Supplementary Table 9. Number of groups of orthologous proteins found exclusively in the selected *Mycobacterium tuberculosis* genomes.

| Name (accession number)† | Number of orthologous groups | RD |
| --- | --- | --- |
| Hyphotetical or PE/PPE proteins | 15 | 12 in RD4 |
| GDP-D-mannose dehydratase GmdA (Rv1511) | 1 | RD4 |
| Nucleotide-sugar epimerase EpiA (Rv1512) | 1 | RD4 |
| Anti-anti-sigma factor RsfB (Rv3687c) | 1 | * |
| 6-phosphogluconate dehydrogenase Gnd1 (Rv1844c) | 1 | * |
| Cytochrome P450 (Rv1256c; Rv3518c*; Rv3121) | 3 | RD12 (Rv3121) and RD13 (Rv1256c) |
| Transcriptional regulator (Rv1255c) | 1 | RD13 |
| ESAT-6 EsxF (Rv3905c) | 1 | * |
| Prophage proteins (Rv2655c; Rv2658c; Rv2659c) | 3 | RD11 |
| Mce family (Rv1971; Rv1966; Rv1969; Rv1970; Rv1967; Rv0176; Rv1973) | 7 | RD7 |
| Oxidoreductase (Rv2073c) | 1 | RD9 |
| Pyridoxine/pyridoxamine 5'-phosphate oxidase (Rv1155) | 1 | * |
| Total of groups of orthologous genes | 36 | - |

† *M. tuberculosis* H37Rv access number; PE/PPE: proline-glutamic and proline-proline-glutamic; RD = region of difference. * = possible pseudogenes.
