# Supplementary material for: Complete Genome Sequencing of Mycobacterium bovis SP38 and Comparative Genomics of Mycobacterium bovis and M. tuberculosis Strains
Source: Front Microbiol. 2017 Dec 5;8:2389. doi: 10.3389/fmicb.2017.02389 (PMC5723337; doi:10.3389/fmicb.2017.02389)
Supplement: Supplementary file 10 [file Table10.DOCX]

Supplementary Table 10. Polymorphic sites of *Mycobacterium bovis* genomes categorized according to COGs (Cluster of Orthologous Groups).

| COGs | Polymorphic sites | Synonymous | Nonsynonymous |
| --- | --- | --- | --- |
| Cell cycle control, cell division, chromosome partitioning | 21 (0.60%) | 10 (0.60%) | 11 (0.61%) |
| Cell wall/membrane/envelope biogenesis | 81 (2.34%) | 34 (2.05%) | 47 (2.62%) |
| Cell motility | 12 (0.34%) | 6 (0.36%) | 6 (0.33%) |
| Post-translational modification, protein turnover, and chaperones | 73 (2.11%) | 30 (1.81%) | 43 (2.40%) |
| Signal transduction mechanisms | 102 (2.95%) | 42 (2.53%) | 60 (2.35%) |
| Intracellular trafficking, secretion, and vesicular transport | 8 (0.23%) | 6 (0.36%) | 2 (0.11%) |
| Defense mechanisms | 52 (1.50%) | 17 (1.02%) | 35 (1.95%) |
| Extracellular structures | 1 (0.03%) | 1 (0.06%) | 0 (0.00%) |
| Mobilome: transposons and prophages | 37 (1.07%) | 14 (0.84%) | 23 (1.28%) |
| RNA processing and modification | 3 (0.08%) | 2 (0.12%) | 1 (0.05%) |
| Translation, ribosomal structure and biogenesis | 84 (2.43%) | 34 (2.05%) | 50 (2.79%) |
| Transcription | 119 (3.45%) | 58 (3.50%) | 61 (3.40%) |
| Replication, recombination and repair | 113 (3.27%) | 39 (2.35%) | 74 (4.13%) |
| Energy production and conversion | 157 (4.55%) | 57 (3.43%) | 100 (5.58%) |
| Amino acid transport and metabolism | 137 (3.97%) | 48 (2.89%) | 83 (4.63%) |
| Nucleotide transport and metabolism | 46 (1.33%) | 20 (1.20%) | 23 (1.28%) |
| Carbohydrate transport and metabolism | 97 (2.81%) | 34 (2.05%) | 63 (3.52%) |
| Coenzyme transport and metabolism | 106 (3.07%) | 42 (2.53%) | 64 (3.57%) |
| Lipid transport and metabolism | 173 (5.01%) | 65 (3.92%) | 108 (6.03%) |
| Inorganic ion transport and metabolism | 94 (2.72%) | 35 (2.11%) | 59 (3.29%) |
| Secondary metabolites biosynthesis, transport, and catabolism | 218 (6.32%) | 77 (4.64%) | 141 (7.87%) |
| General function prediction only | 280 (8.12%) | 94 (5.67%) | 186 (10.39%) |
| Function unknown | 1,093 (31.70%) | 364 (21.95%) | 729 (40.72%) |
| Polymorphic sites in CDSs | 2,804 (81.32%) | 1,014 (61.15%) | 1,790 (100.00%) |
| Total of polymorphic sites | 3,448 | 1,658 (48.08%) | 1,790 (51.92%) |

One protein can be categorized with more than one COG
