# Supplementary material for: Complete Genome Sequencing of Mycobacterium bovis SP38 and Comparative Genomics of Mycobacterium bovis and M. tuberculosis Strains
Source: Front Microbiol. 2017 Dec 5;8:2389. doi: 10.3389/fmicb.2017.02389 (PMC5723337; doi:10.3389/fmicb.2017.02389)
Supplement: Supplementary file 11 [file Table11.DOCX]

Supplementary Table 11. Polymorphic sites of *Mycobacterium tuberculosis* genomes categorized according to COGs (Cluster of Orthologous Groups).

| COGs | Polymorphic sites | Synonymous | Nonsynonymous |
| --- | --- | --- | --- |
| Cell cycle control, cell division, chromosome partitioning | 37 (0.44%) | 13 (0.38%) | 24 (0.48%) |
| Cell wall/membrane/envelope biogenesis | 232 (2.78%) | 85 (2.52%) | 147 (2.96%) |
| Cell motility | 37 (0.44%) | 17 (0.50%) | 20 (0.40%) |
| Post-translational modification, protein turnover, and chaperones | 157 (1.88%) | 68 (2.01%) | 89 (1.80%) |
| Signal transduction mechanisms | 213 (2.55%) | 82 (2.43%) | 131 (2.64%) |
| Intracellular trafficking, secretion, and vesicular transport | 21 (0.25%) | 12 (0.35%) | 9 (0.18%) |
| Defense mechanisms | 156 (1.87%) | 67 (1.98%) | 89 (1.80%) |
| Extracellular structures | 3 (0.03%) | 1 (0.03%) | 2 (0.04%) |
| Mobilome: transposons and prophages | 92 (1.10%) | 37 (1.09%) | 55 (1.10%) |
| Translation, ribosomal structure and biogenesis | 230 (2.76%) | 83 (2.46%) | 120 (2.41%) |
| Transcription | 255 (3.06%) | 99 (2.93%) | 156 (3.14%) |
| Replication, recombination and repair | 203 (2.43%) | 83 (2.46%) | 120 (2.41%) |
| Energy production and conversion | 367 (4.40%) | 156 (4.62%) | 205 (4.13%) |
| Amino acid transport and metabolism | 281 (3.37%) | 110 (3.26%) | 171 (3.44%) |
| Nucleotide transport and metabolism | 74 (0.88%) | 36 (1.06%) | 38 (0.76%) |
| Carbohydrate transport and metabolism | 217 (2.60%) | 82 (2.43%) | 135 (2.72%) |
| Coenzyme transport and metabolism | 227 (2.72%) | 90 (2.66%) | 137 (2.76%) |
| Lipid transport and metabolism | 415 (4.98%) | 185 (5.48%) | 240 (4.83%) |
| Inorganic ion transport and metabolism | 252 (3.02%) | 87 (2.57%) | 165 (3.32%) |
| Secondary metabolites biosynthesis, transport, and catabolism | 450 (5.40%) | 197 (5.83%) | 253 (5.10%) |
| General function prediction only | 486 (5.83%) | 207 (6.13%) | 279 (5.62%) |
| Function unknown | 4,276 (51.30%) | 1,657 (49.11%) | 2,619 (52.80%) |
| Polymorphic sites in CDSs | 8,293 (99.50%) | 3,332 (98.75%) | 4,961 (100.00%) |
| Total of polymorphic sites | 8,335 | 3,374 (40.48%) | 4,961 (59.52%) |

One protein can be categorized with more than one COG
