# Supplementary material for: Complete Genome Sequencing of Mycobacterium bovis SP38 and Comparative Genomics of Mycobacterium bovis and M. tuberculosis Strains
Source: Front Microbiol. 2017 Dec 5;8:2389. doi: 10.3389/fmicb.2017.02389 (PMC5723337; doi:10.3389/fmicb.2017.02389)
Supplement: Supplementary file 12 [file Table12.DOCX]

Supplementary Table 12. Polymorphic sites of *Mycobacterium bovis* BCG genomes categorized according to COGs (Cluster of Orthologous Groups)

| COGs | Polymorphic sites | Synonymous | Nonsynonymous |
| --- | --- | --- | --- |
| Cell cycle control, cell division, chromosome partitioning | 37 (3.4%) | 13 (2.33%) | 24 (4.51%) |
| Post-translational modification, protein turnover, and chaperones | 12 (1.10%) | 5 (0.90%) | 7 (1.31%) |
| Signal transduction mechanisms | 29 (2.66%) | 2 (0.36%) | 25 (4.70%) |
| Intracellular trafficking, secretion, and vesicular transport | 3 (0.29%) | 2 (0.36%) | 1 (0.18%) |
| Defense mechanisms | 1 (0.09%) | 0 (0.00%) | 1 (0.18%) |
| Mobilome: transposons and prophages | 11 (1.01%) | 4 (0.72%) | 7 (1.31%) |
| Translation, ribosomal structure and biogenesis | 19 (1.74%) | 5 (0.90%) | 14 (2.63%) |
| Transcription | 30 (2.75%) | 6 (1.08%) | 24 (4.51%) |
| Replication, recombination and repair | 10 (0.92%) | 1 (0.18%) | 9 (1.70%) |
| Energy production and conversion | 38 (3.49%) | 10 (1.80%) | 28 (5.26%) |
| Amino acid transport and metabolism | 19 (1.74%) | 5 (0.90%) | 14 (2.63%) |
| Nucleotide transport and metabolism | 10 (0.92%) | 2 (0.36%) | 8 (1.50%) |
| Carbohydrate transport and metabolism | 13 (1.19%) | 2 (0.36%) | 11 (2.06%) |
| Coenzyme transport and metabolism | 16 (1.47%) | 2 (0.36%) | 14 (2.63%) |
| Lipid transport and metabolism | 27 (2.48%) | 6 (1.08%) | 21 (3.94%) |
| Inorganic ion transport and metabolism | 16 (1.47%) | 3 (0.54%) | 13 (2.44%) |
| Secondary metabolites biosynthesis, transport, and catabolism | 22 (2.02%) | 6 (1.08%) | 16 (3.00%) |
| General function prediction only | 27 (2.48%) | 12 (2.15%) | 15 (2.82%) |
| Function unknown | 471 (43.30%) | 155 (27.87%) | 316 (59.40%) |
| Polymorphic sites in CDSs | 757 (69.57%) | 225 (40.46%) | 532 (100.00%) |
| Total of polymorphic sites | 1,088 | 556 (51.10%) | 532 (48.90%) |

One protein can be categorized with more than one COG
